# Dysnatremia in COVID-19 Patients—An Analysis of the COLOS Study

**DOI:** 10.3390/jcm12082802

**Published:** 2023-04-10

**Authors:** Anna Królicka, Krzysztof Letachowicz, Barbara Adamik, Adrian Doroszko, Krzysztof Kaliszewski, Katarzyna Kiliś-Pstrusińska, Krzysztof Kujawa, Agnieszka Matera-Witkiewicz, Marcin Madziarski, Michał Pomorski, Marcin Protasiewicz, Janusz Sokołowski, Małgorzata Trocha, Ewa Anita Jankowska, Katarzyna Madziarska

**Affiliations:** 1 Faculty of Medicine, Wroclaw Medical University, Borowska Street 213, 50-556 Wroclaw, Poland; 2 Clinical Department of Nephrology and Transplantation Medicine, Wroclaw Medical University, Borowska Street 213, 50-556 Wroclaw, Poland; 3 Clinical Department of Anaesthesiology and Intensive Therapy, Wroclaw Medical University, Borowska Street 213, 50-556 Wroclaw, Poland; 4 Clinical Department of Internal and Occupational Diseases, Hypertension and Clinical Oncology, Wroclaw Medical University, Borowska 213, 50-556 Wroclaw, Poland; 5 Clinical Department of General, Minimally Invasive and Endocrine Surgery, Wroclaw Medical University, Borowska Street 213, 50-556 Wroclaw, Poland; 6 Clinical Department of Pediatric Nephrology, Wroclaw Medical University, Borowska Street 213, 50-556 Wroclaw, Poland; 7 Statistical Analysis Centre, Wroclaw Medical University, K. Marcinkowski Street 2-6, 50-368 Wroclaw, Poland; 8 Screening of Biological Activity Assays and Collection of Biological Material Laboratory, Wroclaw Medical University Biobank, Wroclaw Medical University, Borowska Street 211A, 50-556 Wroclaw, Poland; 9 Clinical Department of Rheumatology and Internal Medicine, University Hospital, Borowska Street 213, 50-556 Wroclaw, Poland; 10 Clinical Department of Gynecology and Obstetrics, Wroclaw Medical University, Borowska Street 213, 50-556 Wroclaw, Poland; 11 Clinical Department of Cardiology, Wroclaw Medical University, Borowska Street 213, 50-556 Wroclaw, Poland; 12 Clinical Department of Emergency Medicine, Wroclaw Medical University, Borowska Street 213, 50-556 Wroclaw, Poland; 13 Department of Pharmacology, Wroclaw Medical University, Mikulicz-Radecki Street 2, 50-345 Wroclaw, Poland; 14 Institute of Heart Diseases, University Hospital, Borowska Street 213, 50-556 Wroclaw, Poland

**Keywords:** hyponatremia, hypernatremia, COVID-19, ICU admission, mortality

## Abstract

Background: Sodium imbalance is one of the most common electrolyte disturbances encountered in the medical practice, and it may present with either hyponatremia or hypernatremia. Both sodium abnormalities are related with unfavorable outcomes. Objective: Elucidation of the prevalence of dysnatremia among COVID-19 patients and its impact on 30- and 90-day mortality and need for ICU admission was the goal. Design and participants: A single-center, retrospective, observational study was conducted. A total of 2026 adult, SARS-CoV-2 positive patients, admitted to Wroclaw University Hospital between 02.2020 and 06.2021, were included. On admission, patients were divided into groups: normonatremic (N), hyponatremic (L), and hypernatremic (H). Acquired data was processed, and Cox hazards regression and logistic regression were implemented. Key results: Hyponatremia on admission occurred in 17.47% (*n* = 354) of patients and hypernatremia occurred in 5.03% (*n* = 102). Dysnatremic patients presented with more comorbidities, used more drugs, and were statistically more often admitted to the ICU. Level of consciousness was the strongest predictor of ICU admission (OR = 1.21, CI: 1.16–1.27, *p* < 0.001). Thirty-day mortality was significantly higher in both the L and H groups (28.52%, *p* = 0.0001 and 47.95%, *p* < 0.0001, respectively), in comparison to 17.67% in the N group. Ninety-day mortality showed a similar trend in all study groups: 34.37% in the L group (*p* = 0.0001), 60.27% (*p* < 0.0001) in the H group, and 23.32% in the N group. In multivariable analyses, hypo- and hypernatremia were found to be independent predictors of 30- and 90-day mortality. Conclusions: Both hypo- and hypernatremia are strong predictors of mortality and disease severity in COVID-19 patients. Extraordinary care should be taken when dealing with hypernatremic, COVID-positive patients, as this group exhibits the highest mortality rates.

## 1. Introduction

Sodium imbalance is one of the most common electrolyte disturbances encountered in the medical practice and may present with either hyponatremia or hypernatremia. Hyponatremia is defined as serum sodium below 135 mEq/L. The etiology of hyponatremia often remains unclear. Apart from systemic disorders, it has been observed in a variety of infectious diseases. Studies show that hyponatremia is associated with prolonged length of in-hospital stay, higher risk of hospital readmission, significantly higher mean hospital cost, and higher in-hospital as well as overall mortality. Moreover, it has been proved that correction of previously lowered serum sodium could reduce the length of hospital stay, costs, and all-cause mortality risk [1]. Hypernatremia is defined as serum sodium above 145 mmol/L. The most common causes of hypernatremia are loss of pure water and/or hypotonic fluids, excessive salt intake, and hypodipsia [2]. Hypernatremia in the ICU is associated with poorer outcomes, including greater mortality and prolonged in-hospital stays [3]. Correction of sodium level, slow as well as rapid, is associated with better clinical outcomes [4]. The aim of this study was to assess the prevalence of dysnatremia among COVID-19 patients and to elucidate its impact on patients’ outcomes.

## 2. Materials and Methods

### 2.1. Study Design

A single-center, retrospective, observational study was carried out. A total of 2026 patients who were at least 18 years old, had a laboratory-confirmed SARS-CoV-2, and were admitted to any of hospital wards including the emergency department between February 2020 and June 2021, were included. The study protocol for the COLOS (COronavirus in the LOwer Silesia registry) study has been approved by the Institutional Review Board and Ethics Committee at the Wroclaw Medical University, Wroclaw, Poland (No.: KB-444/2021). The Bioethics Committee approved the publication of fully anonymized data. Written informed consent to participate in the study was waived. Dysnatremia was defined as serum sodium below 135 mmol/L (hyponatremia) or above 145 mmol/L (hypernatremia). The COVID-19 disease was diagnosed using RT-PCR assay of a specimen collected via nasopharyngeal swab.

The patients were divided into three groups, depending on natremia on admission: normonatremic (N group), hyponatremic (L group) and hypernatremic (H group).

### 2.2. Data Collection

The dataset was obtained from patients’ charts and included demographics, comorbidities, procedures, vital signs, and laboratory measurements during hospitalization. In this study, the following variables were included: age, gender, heart rate (HR) and blood pressure, consciousness (a zero-one classification: preserved/impaired consciousness as part of triage of patients), as well as comorbidities, namely hypertension, history of myocardial infarction, history of stroke, diabetes mellitus (DM), chronic kidney disease (CKD), heart failure (HF), chronic obstructive pulmonary disease (COPD) or asthma, peripheral occlusive arterial disease (POAD), history of stroke or transient ischemic attack (TIA), and history of neoplastic disorder. Laboratory measurements were also considered, namely white blood cell (WBC) and platelet counts, hemoglobin, creatinine, C-reactive protein (CRP), and sodium concentrations, along with preadmission treatment, including angiotensin converting enzyme inhibitors (ACEIs), aldosterone receptor blockers (ARBs), beta-blockers, calcium blockers, mineral receptor antagonists (MRAs), diuretics, statins, acetylsalicylic acid (ASA), anticoagulants, steroids, or immunosuppressants. Procedures performed during hospitalization, such as ICU admission, respiratory support, use of catecholamine, intravenous (IV) loop diuretic, start of renal replacement therapy (RRT), steroids, and antibiotics were also considered. Cardiovascular disease was defined as HF, PAOD, and a history of myocardial infarction or stroke.

### 2.3. Follow Up and Outcomes

The follow–up period started from the day of admission and ended on the day of discharge or death. The entire hospitalization was analyzed. Further information regarding the patients’ deaths was collected after 90 days after the admission. Primary outcomes were 30-day and 90-day mortality. Secondary outcomes were the need for respiratory support, use of catechol amines, ICU admission, use of steroids, use of antibiotics, use of IV loop diuretics, and need for RRT.

### 2.4. Statistical Analysis

Data was presented either using the mean with SD, median with IQR, or with frequencies and percentages. Due to the large sample size, the normal data distribution was not checked. Instead, the between-group differences in the variance were verified with the use of the Brown–Forsythe test. Quantitative variables were tested by the analysis of variance (ANOVA or Welch’s ANOVA for equal and unequal variances, respectively), and Tukey’s post hoc test. The differences in occurrence frequency (for qualitative variables) were tested by the chi-square test. The *p*-value of <0.05 was considered significant. In each patient group (N, L, H) a univariate and multivariate analysis was carried out. Variables with a *p* < 0.05 in the univariate analysis were entered into the regression model. In each of the N, L, and H groups, 3 regression models were tested: logistic regression considering the prediction of sodium levels, Cox regression for recognizing survival predictors (30-day and 90-day), and logistic regression for assessing the significance of the predictors on the need for treatment in the ICU. In the multivariate logistic regression, the best subset selection model was performed with the use of the MuMIn R-package (Bartoń 2020). The models have been ranked with the use of the Akaike information criterion (AIC), and the best models were averaged according to Akaike weights.

For survival analyses, we generated Kaplan–Meier curves, the comparison of which was performed using the log-rank test. For the control of the Type I error, the Bonferroni correction was applied to the *p*-values of log-rank test results. The significance of the survival time predictors was estimated using the Cox regression. Statistical analysis was performed with the use of the Statistica (Tibco Software Inc., Palo Alto, CA, USA, 2017) and R package “MuMIn” (Bartoń 2020) run in the R environment v. 4.1.2 (R Core Team 2021).

## 3. Results

### 3.1. Patient Characteristics

Patient characteristics alongside with a comparison of the N, L, and H patient groups are presented in Table 1. Median age of all patients was 65 (IQR: 48.0–74.0), while 50.4% were male and 49.6% were female. On admission, 17.47% of patients were hyponatremic and 5.03% were hypernatremic. Mean sodium level equaled 130.39 ± 5.27 in the L group, 150.14 ± 5.45 in the H group, and 139.04 ± 2.55 in the N group. Among vital signs, SBP was statistically lower in the H group than in the N and L groups (121.13 ± 26.72 vs. 132.33 ± 22.46 *p* = 0.0000 and 134.28 ± 23.13. *p* = 0.0000, respectively). Dysnatremic patients had more comorbidities than normonatremic patients. They suffered more often from hypertension, HF, DM and had myocardial infarction histories (Table 1). Long-term use of drugs was also more common among dysnatremic patients, with statistical difference considering ARNI/ARB/ACEI, beta-blocker, diuretic, and anticoagulant use. Among laboratory findings, dysnatremic patients had significantly higher creatinine and CRP values on admission (Table 1).

Among all 2026 patients, 212 (10.5%) were admitted to the ICU, 383 (18.9%) needed respiratory support, 217 (10.7%) needed vasopressors, 333 (16.4%) needed IV loop diuretics, and in 72 (3.6%) of the cases RRT had to be started. Steroids were used for 1090 patients (53.8%), and antibiotics for 1229 (60.7%) patients. Dysnatremic patients were statistically more often admitted to the ICU, with 13.0% in the L group and 21.6% in the H group vs. 9.2% in the N group. *p* = 0.0001. They also needed respiratory support more often, with 21.3% in the L group and 34.3% in the H group vs. 17.4% in the N group, *p* = 0.0001. IV loop diuretics were used more commonly in the L and H groups, in comparison to the N group, with 20.3% and 31.4% of patients vs. 14.65 *p* = 0.0000, respectively. RRT was started more frequently in dysnatremic patients, with 14.8% in the L group and 9.8% in the H group vs. 2.9% in the N group, *p* = 0.0005.

### 3.2. Univariate Analyses

Univariate analyses were carried out in the hyponatremic and hypernatremic groups, considering mortality predictors and predictors of sodium levels, as well as ICU admission predictors. The results of these analyses are presented in Table 2, Table 3 and Table 4.

### 3.3. Kaplan-Meyer Curve

**Figure 1:**
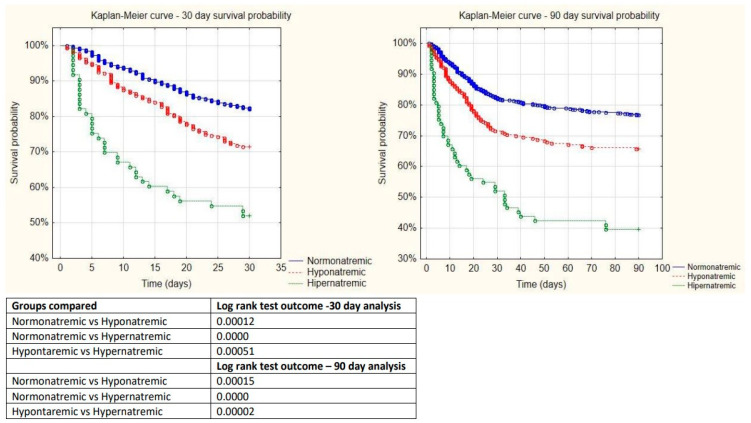
Kaplan–Meyer curves for 30- and 90-day mortality and results of the log-rank tests.

Thirty-day mortality equaled 28.52% in the L group and 47.95% in the H group, compared to 17.67% in the N group, *p* = 0.00036 and *p* = 0.0000, respectively (Figure 1). Ninety-day mortality equaled 34.37% in the L group, 60.27% in the H group, and 23.32% in the N patients, *p* = 0.00045 and *p* = 0.0000, respectively (Figure 1).

### 3.4. 30-Day and 90-Day Mortality Predictors

**Figure 2:**
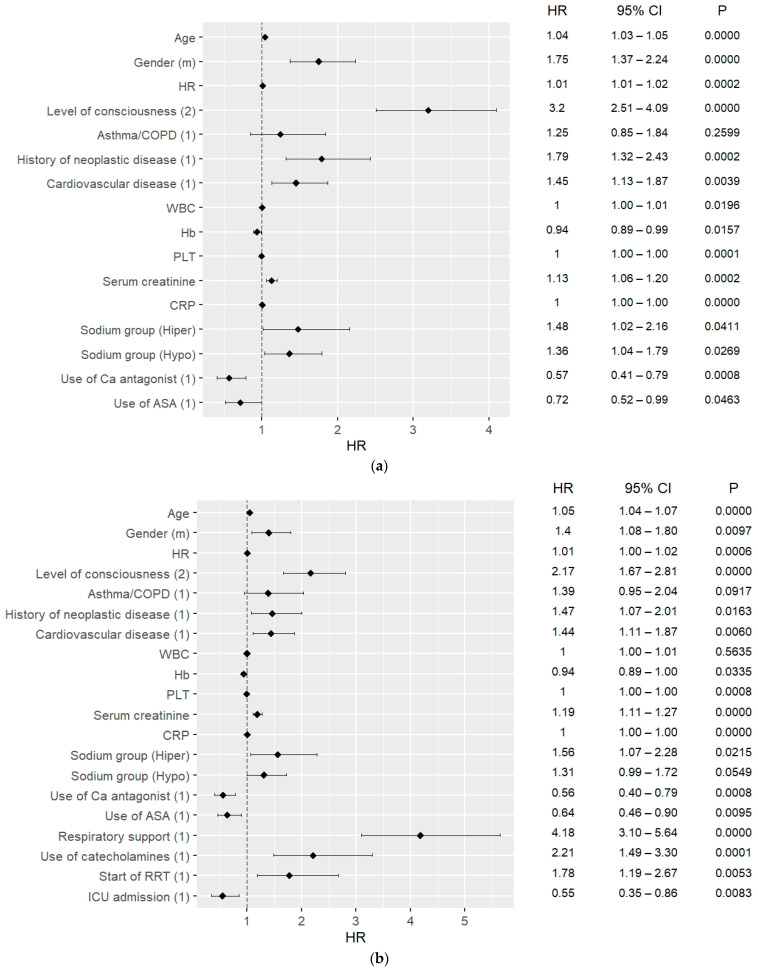

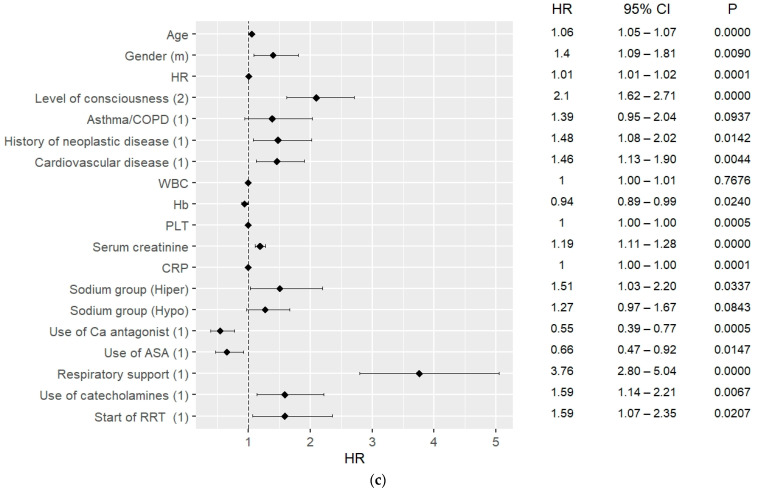
(**a**) 30-day mortality predictors in COVID patients, data on admission; (**b**) 30-day mortality predictors in COVID patients, including ICU patients; (**c**) 30-day mortality predictors in COVID patients, excluding ICU patients.

**Figure 3:**
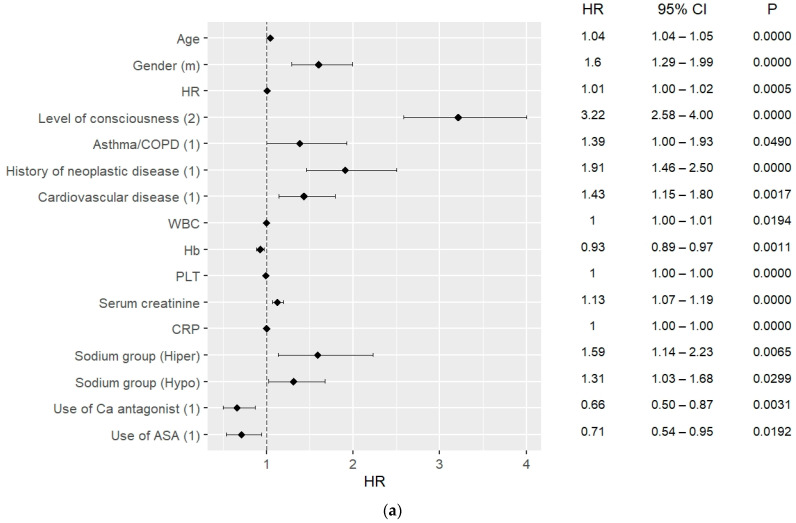

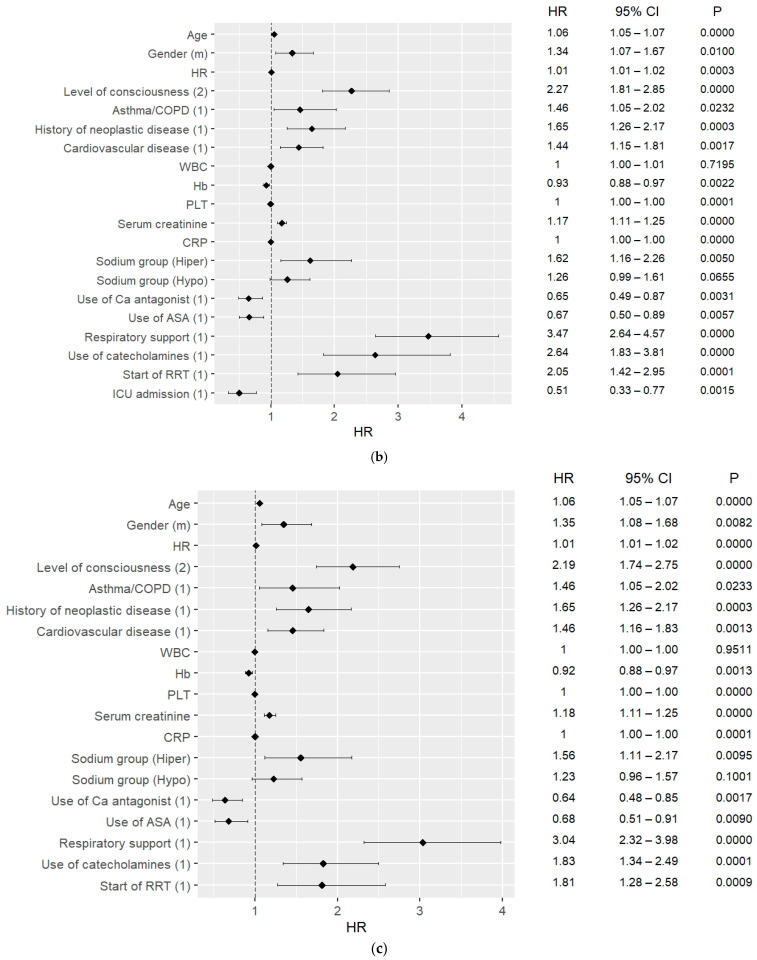
(**a**) 90-day mortality predictors in COVID patients, data on admission; (**b**) 90-day mortality predictors in COVID patients, including ICU patients; (**c**) 90-day mortality predictors in COVID patients, excluding ICU patients.

The results of the multivariate Cox hazard analysis are presented in Figure 2a–c and Figure 3a–c. The analysis was carried out considering 30-day and 90-day mortality, and when including ICU admission and excluding ICU admission.

Hypernatremia was a strong mortality predictor for both 30-day and 90-day mortality, including for ICU patients as well as outside the ICU. For the 30-day mortality, HR = 1.58, CI: 1.13–2.21, *p* = 0.0070 excluding ICU and HR = 1.60, CI: 1.14–2.24, *p* = 0.006 including ICU admissions; for the 90-day mortality, HR = 1.55; CI: 1.11–2.24 *p* = 0.0100 excluding ICU admission, and HR = 1.61; CI: 1.15–2.25 *p* = 0.0050 including ICU admissions. Moreover, hyponatremia on admission could predict 30-day mortality with HR = 1.30, CI: 1.02–1.66, *p* = 0.037.

One of the strongest predictors for both 30- and 90-day mortality was the need for respiratory support, both considering ICU hospitalizations and non-ICU patients. For the 30-day mortality, the need for respiratory support could predict mortality with HR = 3.09, CI: 2.36–4.03, *p* < 0.0010 excluding ICU patients and HR = 3.44, CI: 2.62–4.52, *p* < 0.0010 including ICU patients. For the 90-day mortality, the need for respiratory support could predict mortality with similar values, including as well as excluding ICU patients (Figure 3a,b). Other strong mortality predictors among in-hospital procedures were the use of catecholamines for the 30- and 90-day mortality and start of RRT for the 90-day mortality (Figure 2a–c and Figure 3a–c). ICU admission could predict survival in both 30-day and 90-day analyses, with HR = 0.5, CI: 0.34–0.78, *p* = 0.002 for both.

In the analysis of vital signs, DBP, HR, and level of consciousness were found to be factors predicting mortality, the strongest one being the level of consciousness, with HR = 2.11, CI: 1.68–2.64, *p* < 0.0010 for 30-day mortality outside the ICU.

Considering the comorbidities, the strongest mortality predictor of them was CVD (HR = 2.62, CI: 2.10–3.28, *p* = 0.0000, HR = 2.67; CI: 2.19–3.26, *p* = 0.0000). Furthermore, history of neoplastic disorder had the ability to predict 90-day mortality with HR = 1.91, CI: 1.46–2.50, *p* < 0.001. Diagnosis of asthma/COPD could predict 90-day mortality including ICU admissions with HR = 1.46, CI: 1.05–2.02, *p* = 0.023.

Considering long-term use of drugs, the use of calcium blockers and ASA were mortality predictors in all analyses with HR = 0.69, CI: 0.53–0.91, *p* = 0.009, and HR = 0.66, CI: 0.49–0.88, *p* = 0.004 for 30-day mortality, respectively.

Some of the laboratory findings which were analyzed were also found to predict 30-day and 90-day mortality. These were platelets (HR = 1.00; CI: 1.00–1.00 *p* < 0.001), CRP (HR = 1.00; CI: 1.00–1.00 *p* < 0.001), and creatinine (HR = 1.18; CI: 1.12–1.25 *p* < 0.001) for 30-day mortality. WBC level on admission was also a 30-day mortality predictor (HR = 1.01; CI: 1.00–1.01 *p* = 0.015). The level of hemoglobin was found to predict 30-day mortality with HR = 0.92, CI: 0.88–0.97, *p* < 0.0010 including ICU admissions, HR = 0.93, CI: 0.89–0.97, *p* = 0.0010 excluding ICU admissions, and 90-day mortality with similar values (Figure 3b,c).

### 3.5. ICU Admission Predictors

Univariate and multivariate analysis of predictors of ICU admission are presented in Table 3. Among the vital signs, level of consciousness was the strongest predictor of ICU admission (OR = 1.2050, CI: 1.1558–1.2564, *p* = 0.0000). WBC and CRP concentration were also able to predict ICU admission, with OR = 1.0037, CI: 1.0024–1.0050, *p* = 0.0000 and OR = 1.0005, CI: 1.0003–1.0007, *p* = 0.0000, respectively.

### 3.6. Possible Dysnatremia Predictors

The results of the multivariate analysis revealed possible predictors of hyponatremia and hypernatremia, which are presented in Table 5. Low sodium level was strongly associated with diagnosis of DM and intake of ACEI/ARB/ARNI, with OR = 1.40, CI: 1.02–1.93, *p* = 0.0376 and OR = 1.52, CI: 1.08–2.15, *p* = 0.0170, respectively. Impaired consciousness was very strongly correlated with hypernatremia, with OR = 8.54, CI: 4.83–15.09, *p* = 0.0000, alongside SBP and CRP (Table 5).

## 4. Discussion

Our analysis of 2026 COVID-19 positive patients, admitted to the Wroclaw University Hospital, revealed that sodium imbalance is not a rare finding and influences survival. The prevalence of hyponatremia (17.47%) and hypernatremia (5.03%) in our study is coherent with previous studies, in which hyponatremia is reported to occur in 9.9–35.8% and hypernatremia in 2.4–5.3% of COVID-19 positive inpatients [5]. In our study, a majority of the admitted patients suffered from mild-to-moderate hyponatremia and hypernatremia. According to Ruiz-Sánchez, J.G. et al., dysnatremic patients in the HOPE study suffered statistically more often from hypertension, CKD, DM, and CVD [6]. Our findings are partially consistent with the mentioned paper, as in our cohort dysnatremic patients suffered more often from hypertension, HF, DM, and had myocardial infarction histories. The differences between dysnatremic and normonatremic groups regarding long-term intake of drugs seem to be in line with the underlying comorbidities. Mainly, ACEI/ARB/ARNI, beta-blockers, and diuretics are used in the treatment of hypertension, HF, and CVD.

In our study, both hypo- and hypernatremia were found to be mortality predictors. It is important to remember that hyponatremia and hypernatremia are themselves pathophysiologic processes indicating disturbed water homeostasis [7]. Both 30- and 90-day mortality rates among hypo- and hypernatremic patients were significantly higher than in normonatremic patients. Early recognition of dysnatremia is a strong warning for the fatal course of disease, which is why physicians should exhibit extra caution with such patients. Here, a question on the possible correction of dysnatremia on admission arises. Some studies show that correction of hyponatremia results in a reduction in overall mortality in this patient group [8]. However, Chewcharat A. et al., have proved that both patients with uncorrected as well as corrected hyponatremia have significantly higher, and most importantly similar, in-hospital and 1-year mortality rates compared to normonatremic patients [9]. To our knowledge, there are no randomized trials on the correction of dysnatremia in COVID-19 patients and its impact on illness severity, outcomes, and mortality. In one retrospective study, de La Flor, J.C. et al., found that the absence of correction of hyponatremia in the first 72–96 h is associated with higher mortality in COVID-19 patients [10]. According to a recent study carried out in the Wroclaw Medical University Hospital, sodium concentration could predict prolonged length of in-hospital stay in patients with atrial fibrillation [11].

Another important and easily detected mortality predictor was found to be impaired level of consciousness, which is coherent with previous studies [12]. COVID-19 positive patients may present with a number of neurological symptoms, such as seizures, altered mentation with normal imaging, or a neuro-COVID-19 complex [12]. The most common manifestation is reported to be altered mentation, which in 23.6% of cases has no reasonable explanation [12]. Therefore, impaired consciousness is an indicator of illness severity and should raise clinicians’ concerns.

It has been broadly proven, that many common preexisting comorbidities, such as hypertension, DM, CVD, COPD, and CKD, are mortality predictors in COVID-19 patients [13]. Our results are partially consistent with these findings, as in our cohort history of neoplastic disease, CVD, and asthma/COPD could predict mortality. Long-term use of certain drugs, such as calcium blockers or ASA, being either a 30-day or 90-day mortality predictor could be explained by the fact that these drugs are used in the treatment of certain medical conditions, such as hypertension or CVD [14,15]. These conditions were listed above as they themselves are COVID-19 mortality predictors. The dilemma on whether the renin–angiotensin system blockade could worsen the patients’ outcomes and whether it should be discontinued after admission rose in the early phase of the pandemic. Our study shows no correlation between the long-term use of ACEI/ARNI/ARB and increased mortality as a result of possible increased patients’ susceptibility to SARS-CoV infection. A metanalysis of observational studies suggests that a therapy aimed at blocking the RAA system should not be discontinued; however, randomized trials are crucial for confirmation of this theory [16].

Another important finding in our study was the establishment of predictors of ICU admission. Impaired consciousness was found to strongly predict ICU admission, along with WBC and CRP.

Regarding laboratory findings, WBC was able to predict both increased mortality as well as ICU admission, while CRP was an ICU admission predictor. Both WBC and CRP are signs of possible bacterial co-infection, which is known to be a COVID-19 complication occurring more often in critically ill patients [17]. Therefore, higher WBC values should draw clinicians’ attention, as they may suggest fatal outcomes in COVID-19 patients. It has been previously reported that the degree of lymphopenia as well as increased neutrophil levels may be used to predict illness severity in COVID-19 patients [13].

Our study has some strengths and limitations. The main drawback of the study is the lack of measurement of patients’ osmolarity/tonicity profile, and, therefore, a lack of diagnosis of mechanism of the underlying dysnatremia. Furthermore, the observation of the influence of impaired consciousness on fluid intake is missing. In this study, dysnatremia is solely a laboratory finding indicating severe outcomes. Osmolarity measurement was impossible to carry out, due to the retrospective character of the study. However, the study analyses a huge patient probe, namely 2026 individuals. We identified predictors of 30- and 90-day mortality, dysnatremia, and ICU admission, which are quick and easy to implement in daily practice, as well as inexpensive.

## 5. Conclusions

Dysnatremia is a common finding in COVID-19 patients. Hyponatremia occurs more often than hypernatremia; however, hypernatremia is found to be more severe and fatal. Both hypo- and hypernatremia are strong mortality predictors in COVID-19 patients. Impaired level of consciousness as well as high WBC levels are strong predictors of both increased mortality and ICU admission. Hypernatremia was strongly associated with impaired level of consciousness, while hyponatremia was associated with a diagnosis of DM and ACEI/ARB/ARNI intake. Patients suffering from dysnatremia should be recognized early as they require more meticulous care.

## Figures and Tables

**Table 1:** Patient characteristics.

Characteristic	All	Normonatremia	Hyponatremia	Hypernatremia	*p*-Value (ANOVA/Chi Square)	Hypo vs. Normo	Hyper vs. Normo	Hypo vs. Hyper
Mean ± SD; median (Q1–Q3) or *n* (%)		1570 (77.49%)	354 (17.47%)	102 (5.03%)				
Age, yr.	61.21 ± 18.21	59.34 ± 18.55	66.70 ± 15.10	70.96 ± 15.86	0.0000 ^w^	0.0000	0.0000	0.2036
	65.0 (48.0–74.0)	63.00 (45.00–73.00)	68.00 (61.00–77.00)	70.50 (63.00–83.00)				
Gender								
Male	1021 (50.4%)	767 (48.9%)	197 (55.6%)	57 (55.9%)	0.0363			
Female	1005 (49.6%)	803 (51.1%)	157 (44.4%)	45 (44.1%)				
Vital signs								
Systolic BP, mm Hg	132.10 ± 22.93	132.33 ± 22.46	134.28 ± 23.13	121.13 ± 26.72	0.0000	0.2033	0.0000	0.0000
	130.0 (120.0–145.0)	130.0 (120.0–144.0)	130.0 (120.0–150.0)	122.5 (110.0–134.0)				
Diastolic BP	78.02 ± 13.41	78.21 ± 13.10	78.11 ± 13.07	74.54 ± 18.51	0.2389 ^w^			
	80.0 (70.0–85.0)	80.0 (70.0–85.0)	80.0 (70.0–85.0)	72.5 (60.0–85.5)				
Heart rate, BPM	85.64 ± 16.38	84.97 ± 15.71	87.53 ± 17.14	90.04 ± 22.39	0.0164 ^w^	0.0198	0.0075	0.2300
	82.0 (75.0–94.0)	81.0 (75.0–92.0)	83.0 (76.0–96.0)	82.0 (74.0–105.0)				
Impaired consciousness	323 (15.6%)	197 (12.56%)	60 (16.95%)	66 (64.71%)	0.0000			
Comorbidities								
Hypertension	988 (48.8%)	730 (46.5%)	204 (57.6%)	54 (52.9%)	0.0005			
History of myocardial infarction	189 (9.3%)	134 (8.5%)	47 (13.3%)	8 (7.8%)	0.0187			
Heart failure	250 (12.3%)	174 (11.1%)	59 (16.7%)	17 (16.7%)	0.0061			
History of stroke or TIA	160 (7.9%)	116 (7.4%)	32 (9.0%)	12 (11.8%)	0.1929			
Diabetes mellitus	467 (23.1%)	331 (21.1%)	112 (31.7%)	24 (23.5%)	0.0001			
Chronic kidney disease	226 (11.2%)	165 (10.5%)	47 (13.3%)	14 (13.7%)	0.2290			
Chronic obstructive pulmonary disease or asthma	148 (7.3%)	110 (7.0%)	28 (7.9%)	10 (9.8%)	0.5121			
Peripheral occlusive arterial disease	99 (4.9%)	69 (4.4%)	24 (6.8%)	6 (5.9%)	0.1523			
History of cancer	196 (9.7%)	147 (9.4%)	37 (10.5%)	12 (11.8%)	0.6284			
Charlson Comorbidity Index	3.25 ± 2.69	3.00 ± 2.65	3.98 ± 2.65	4.45 ± 2.64	0.0000			
	3(1–5)	3 (1–5)	4(2–5)	4(2–6)				
Drugs								
ACEI/ARB/ARNI	492 (24.3%)	355 (22.6%)	112 (31.6%)	25 (24.5%)	0.0017			
Beta-blockers	522 (25.8%)	383 (24.4%)	111 (31.4%)	28 (27.5%)	0.0238			
Calcium blockers	289 (14.3%)	217 (13.8%)	55 (15.5%)	17 (16.7%)	0.5484			
Mineral receptor antagonists	100 (4.9%)	74 (4.7%)	24 (6.8%)	2 (2.0%)	0.0976			
Diuretics	317 (15.7%)	226 (14.4%)	76 (21.5%)	15 (14.7%)	0.0040			
Statins	349 (17.2%)	270 (17.2%)	59 (16.7%)	20 (19.6%)	0.7849			
Acetylsalicylic acid	254 (12.5%)	195 (12.4%)	47 (13.3%)	12 (11.8%)	0.8818			
Anticoagulants	282 (13.9%)	203 (12.9%)	58 (16.4%)	21 (20.6%)	0.0323			
Steroids or immunosuppressants	119 (5.9%)	99 (6.3%)	18 (5.1%)	2 (2.0%)	0.1531			
Laboratory values								
White blood cell count, 103/mL	9.14 ± 11.72	8.98 ± 12.75	8.88 ± 6.57	12.45 ± 8.19	0.0135	0.8798	0.0038	0.0067
	7.36 (5.37–10.30)	7.21 (5.30–9.99)	7.30 (5.17–10.77)	10.59 (7.82–14.88)				
Hemoglobin, g/dL	12.93 ± 2.29	12.94 ± 2.25	12.84 ± 2.43	12.98 ± 2.42	0.7570 ^w^			
	13.10 (11.70–14.50)	13.20 (11.90–14.40)	13.05 (11.20–14.7)	13.15 (11.40–14.20)				
Platelets, 103/L	232.17 ± 108.40	232.19 ± 107.53	230.32 ± 114.69	238.20 ± 99.85	0.8116			
	211.00 (163.00–282.00)	211.0 (164.0–282.0)	204.5 (160.0–275.0)	229.0 (173.0–300.0)				
Sodium, mEq/L	138.08 ± 5.45	139.04 ± 2.55	130.39 ± 5.27	150.14 ± 5.45	0.0000			
	138.00 (136.00–141.00)	139.0 (137.0–141.0)	132.0 (129.0–134.0)	148.0 (147.0–152.0)				
Serum creatinine, mg/dL	1.32 ± 1.30	1.26 ± 1.22	1.47 ± 1.52	1.70 ± 1.52	0.0017 ^w^	0.0063	0.0008	0.1070
	0.94 (0.76–1.28)	0.92 (0.75–1.22)	1.00 (0.82–1.46)	1.11 (0.83–2.06)				
CRP	78.67 ± 84.77	70.63 ± 78.25	97.96 ± 93.02	133.39 ± 114.57	0.0000 ^w^	0.0000	0.0000	0.0002
	49.28 (13.27–116.40)	43.36 (10.03–105.07)	68.69 (27.52–141.25)	99.05 (46.00–203.60)				
In-hospital procedures								
ICU admission	212 (10.5%)	144 (9.2%)	46 (13.0%)	22 (21.6%)	0.0001			
Respiratory support	383 (18.9%)	273 (17.4%)	75 (21.3%)	35 (34.3%)	0.0001			
No support	887 (43.85%)	736 (46.94%)	131 (37.11%)	20 (19.61%)				
Passive oxygen therapy	753 (37.22%)	559 (35.65%)	147 (41.64%)	47 (46.08%)				
HFNC	131 (6.48%)	99 (6.31%)	22 (6.23%)	10 (9.80%)				
BiPAP/CPAP	42 (2.08%)	27 (1,72%)	13 (3.68%)	2 (1.96%)				
Respiratory therapy	210 (10.38%)	147 (9.38%)	40 (11.33%)	23 (22.55%)				
Vasopressors	217 (10.7%)	147 (9.4%)	41 (11.6%)	29 (28.4%)	0.0000			
Intravenous loop diuretic	333 (16.4%)	229 (14.6%)	72 (20.3%)	32 (31.4%)	0.0000			
Start of renal replacement therapy	72 (3.6%)	45 (2.9%)	17 (14.8%)	10 (9.8%)	0.0005			
Steroids	1090 (53.8%)	834 (53.1%)	194 (54.8%)	62 (60.8%)	0.2958			
Antibiotics	1229 (60.7%)	931 (59.3%)	213 (60.2%)	85 (83.3%)	0.0000			

BP—blood pressure; BPM—beats per minute; TIA—transient ischemic attack; ACEI—angiotensin—converting enzyme inhibitor; ARB—angiotensin II receptor blocker; ARNI—angiotensin receptor—nephrilysin inhibitor; CRP—C reactive protein; ICU—intensive care unit; HFNC—high flow nasal cannula; BiPAP—bilevel positive airway pressure; CPAP—continuous positive airway pressure; ^w^—Welch’s ANOVA.

**Table 2:** Results of the univariate analysis of 30- and 90-day mortality predictors. See the Section 2.4 for details on the multivariate model.

	30-Day Mortality Predictors					90-Day Mortality Predictors				
Characteristics	Hazard Ratio (HR)	Standard Error (SE)	95% HR Lower Bound	95% HR Upper Bound	*p* Value	Hazard Ratio (HR)	Standard Error (SE)	95% HR Lower Bound	95% HR Upper Bound	*p* Value
Hyponatremia	1.69	0.10	1.29	2.21	0.4204	1.60	0.09	1.26	2.04	0.1657
Hypernatremia	3.63	0.12	2.54	5.20	0.0000	3.73	0.11	2.71	5.14	0.0000
Demographic data and vital signs										
Age	1.05	0.00	1.04	1.06	0.0000	1.05	0.00	1.05	1.06	0.0000
Sex (female)	1.54	0.06	1.22	1.94	0.0002	0.73	0.05	0.59	0.89	0.0019
HR	1.01	0.00	1.01	1.02	0.0000	1.01	0.00	1.01	1.02	0.0001
SBP	0.99	0.00	0.99	1.00	0.0153	1.00	0.00	0.99	1.00	0.0448
DBP	0.97	0.00	0.96	0.98	0.0000	0.98	0.00	0.97	0.99	0.0000
Consciousness	5.21	0.06	4.17	6.52	0.0000	5.13	0.05	4.20	6.27	0.0000
Comorbidities										
Hypertension	1.89	0.06	1.49	2.40	0.0000	2.06	0.05	1.67	2.55	0.0000
History of myocardial infarction	2.51	0.07	1.91	3.29	0.0000	2.52	0.06	1.97	3.22	0.0000
History of stroke/TIA	1.60	0.08	1.15	2.23	0.0057	1.73	0.07	1.29	2.30	0.0002
Diabetes mellitus	1.68	0.06	1.33	2.13	0.0000	1.78	0.05	1.45	2.19	0.0000
Chronic kidney disease	2.00	0.07	1.53	2.63	0.0000	2.24	0.06	1.77	2.83	0.0000
Heart failure	2.55	0.06	1.99	3.27	0.0000	2.65	0.06	2.13	3.30	0.0000
Asthma/COBP	1.19	0.10	0.82	1.74	0.3587	1.34	0.08	0.97	1.84	0.0748
Peripheral obstructive arterial disease	2.49	0.09	1.75	3.54	0.0000	2.23	0.08	1.60	3.10	0.0000
History of neoplastic disease	2.40	0.07	1.82	3.17	0.0000	2.50	0.06	1.95	3.21	0.0000
Cardiovascular disease	2.62	0.06	2.10	3.28	0.0000	2.67	0.05	2.19	3.26	0.0000
Long-term use of drugs										
ACEI/ARB/ARNI	1.12	0.06	0.88	1.42	0.3553	1.17	0.05	0.95	1.44	0.1490
Beta-blockers	1.33	0.06	1.06	1.67	0.0152	1.50	0.05	1.23	1.84	0.0001
Calcium antagonists	0.77	0.08	0.56	1.07	0.1162	0.95	0.07	0.73	1.24	0.6906
MRAs	1.24	0.11	0.82	1.88	0.3155	1.56	0.09	1.11	2.19	0.0098
Diuretics	1.45	0.07	1.12	1.87	0.0052	1.55	0.06	1.24	1.95	0.0001
Statins	1.24	0.07	0.96	1.61	0.1055	1.34	0.06	1.07	1.68	0.0115
ASA	0.99	0.08	0.73	1.36	0.9686	1.08	0.07	0.83	1.42	0.5503
Anticoagulants	1.50	0.07	1.15	1.95	0.0030	1.62	0.06	1.29	2.05	0.0000
GCS/immunosupressants	1.09	0.11	0.71	1.68	0.7003	1.19	0.09	0.82	1.73	0.3500
Laboratory findings										
WBC	1.02	0.00	1.02	1.03	0.0000	1.02	0.00	1.02	1.02	0.0000
Hemoglobin	0.87	0.02	0.84	0.91	0.0000	0.87	0.02	0.83	0.90	0.0000
PLT	1.00	0.00	1.00	1.00	0.0063	1.00	0.00	1.00	1.00	0.0024
Creatinine	1.18	0.02	1.12	1.23	0.0000	1.17	0.02	1.13	1.22	0.0000
CRP	1.00	0.00	1.00	1.01	0.0000	1.00	0.00	1.00	1.01	0.0000
Sodium	1.02	0.01	1.00	1.04	0.0635	1.03	0.01	1.01	1.05	0.0082
In-hospital procedures										
ICU admission	3.84	0.06	3.01	4.91	0.0000	3.84	0.06	3.01	4.91	0.0000
Respiratory support	6.05	0.06	4.84	7.58	0.0000	6.05	0.06	4.84	7.58	0.0000
Use of catecholamines	5.60	0.06	4.44	7.06	0.0000	5.60	0.06	4.44	7.06	0.0000
IV loop diuretic	3.93	0.06	3.14	4.91	0.0000	3.93	0.06	3.14	4.91	0.0000
Start of RRT	3.98	0.09	2.81	5.63	0.0000	3.98	0.09	2.81	5.63	0.0000
Use of steroids	1.29	0.06	1.02	1.63	0.0360	1.29	0.06	1.02	1.63	0.0360
Use of antibiotics	3.38	0.08	2.45	4.69	0.0000	3.38	0.08	2.45	4.69	0.0000

HR—heart rate; SBP—systolic blood pressure; DBP—diastolic blood pressure; TIA—transient ischemic attack; COPD—chronic obstructive pulmonary disease; ACEI—angiotensin-converting enzyme inhibitor; ARB—angiotensin II receptor blocker; ARNI—angiotensin receptor-nephrilysin inhibitor; MRA—mineralocorticoid receptor antagonist; ASA—acetylsalicylic acid; GCS—glucocorticoids; WBC—white blood cells count; PLT—platelet count; CRP—C reactive protein; ICU—intensive care unit; IV—intravenous; RRT—renal replacement therapy.

**Table 3:** Results of the univariate and multivariate analysis of ICU admission predictors in COVID-19 patients.

				Univariate Analysis				Multivariate Analysis—Averaged Best Subset Selection Models (with the Use of AIC)			
Characteristics				OR	CI 95 Lower	CI 95 Upper	*p*	OR	CI 95 Lower	CI 95 Upper	*p*
Basic demographic data and vital signs											
Age				0.99	0.99	1.00	0.1877				
Sex (M)				1.94	1.38	2.72	0.0001	1.0550	1.0231	1.0878	0.00063
HR				1.00	0.99	1.01	0.7279				
SBP				0.99	0.98	1.00	0.0099	1.0014	1.0005	1.0023	0.00183
DBP				0.95	0.94	0.96	0.0000	0.9949	0.9934	0.9964	0.00000
Consciousness				0.18	0.13	0.25	0.0000	1.2050	1.1558	1.2564	0.00000
Comorbidities											
Hypertension				1.28	0.92	1.76	0.1380				
Diabetes mellitus				1.39	0.98	1.97	0.0626				
Chronic kidney disease				0.86	0.52	1.40	0.5366				
Asthma/COPD				1.04	0.59	1.82	0.9022				
History of neoplastic disorder	0.94	0.55	1.60	0.8170							
Cardiovascular disease	1.05	0.74	1.50	0.7823							
Laboratory findings											
WBC	1.06	1.04	1.08	0.0000	1.0037	1.0024		1.0050		0.00000	
Hemoglobin	0.94	0.88	1.01	0.0733							
PLT	1.00	1.00	1.00	0.1625							
Creatinine	1.03	0.92	1.16	0.6180							
CRP	1.01	1.01	1.01	0.0000	1.0005	1.0003		1.0007		0.00000	
Hyponatremia	1.35	0.90	2.02	0.3598	1.0061	0.9664		1.0474		0.76825	
Hypernatremia	2.79	1.59	4.91	0.0026	1.0020	0.9313		1.0782		0.95654	

HR—heart rate; SBP—systolic blood pressure; DBP—diastolic blood pressure; COPD—chronic obstructive pulmonary disease; WBC—white blood cells count; PLT—platelet count; CRP—C reactive protein.

**Table 4:** Results of univariate analysis, predictors of hyponatremia and hypernatremia.

Characteristic	Hyponatremia				Hypernatremia			
	OR	CI 95 Upper	CI 95 Lower	*p*	OR	CI 95 Upper	CI 95 Lower	*p*
Age	1.02	1.01	1.03	0.0000	1.04	1.02	1.05	0.0000
Sex (female)	0.86	0.66	1.14	0.2978	0.90	0.56	1.44	0.6510
HR	1.01	1.00	1.02	0.0182	1.01	1.00	1.03	0.0894
SBP	1.00	0.99	1.01	0.8926	0.98	0.97	0.99	0.0018
DBP	1.00	0.99	1.01	0.5917	0.98	0.96	1.00	0.0294
Consciousness	1.49	1.05	2.10	0.0248	11.20	6.72	18.66	0.0000
Comorbidities								
Hypertension	1.40	1.06	1.85	0.0170	1.24	0.77	1.99	0.3854
MI	1.76	1.19	2.59	0.0043	1.13	0.53	2.43	0.7452
Stroke/TIA	1.08	0.67	1.74	0.7530	1.94	0.99	3.81	0.0542
DM	1.71	1.28	2.30	0.0003	1.01	0.58	1.77	0.9712
CKD	1.30	0.89	1.90	0.1714	0.61	0.26	1.43	0.2574
Heart failure	1.57	1.11	2.23	0.0115	1.37	0.73	2.55	0.3237
Asthma/COBP	0.88	0.53	1.46	0.6150	1.74	0.86	3.49	0.1222
POAD	1.63	0.96	2.77	0.0701	1.42	0.55	3.65	0.4721
CVD	1.58	1.18	2.12	0.0022	1.73	1.05	2.84	0.0313
Neoplastic disorder	1.32	0.87	2.00	0.1982	1.30	0.63	2.68	0.4831
Laboratory findings								
WBC	1.00	0.99	1.01	0.7957	1.01	1.00	1.02	0.0613
Hb	0.99	0.94	1.05	0.7718	1.01	0.91	1.12	0.9051
PLT	1.00	1.00	1.00	0.9114	1.00	1.00	1.00	0.2087
Creatinine	1.12	1.02	1.23	0.0141	1.12	0.97	1.29	0.1215
CRP	1.00	1.00	1.00	0.0003	1.01	1.00	1.01	0.0000
Long-term use of drugs								
ACEI/ARB/ARNI	1.64	1.23	2.18	0.0007	1.26	0.75	2.09	0.3821
Beta-blockers	1.41	1.06	1.87	0.0185	1.05	0.62	1.75	0.8629
Ca blockers	1.16	0.82	1.65	0.4078	1.34	0.74	2.41	0.3362
MRAs	1.27	0.76	2.13	0.3657	0.42	0.10	1.75	0.2353
Diuretics	1.40	1.01	1.94	0.0453	0.92	0.49	1.74	0.7943
Statins	1.06	0.76	1.47	0.7382	1.17	0.67	2.06	0.5757
ASA	1.13	0.78	1.63	0.5209	1.02	0.53	1.98	0.9529
Anticoagulants	1.38	0.98	1.94	0.0677	1.77	1.01	3.08	0.0442
Steroids/immunosuppressants	0.99	0.57	1.71	0.9717	0.39	0.09	1.63	0.1977

HR—heart rate; SBP—systolic blood pressure; DBP—diastolic blood pressure; MI—myocardial infarction; TIA—transient ischemic attack; DM—diabetes mellitus; CKD—chronic kidney disease; COPD—chronic obstructive pulmonary disease; POAD—peripheral occlusive arterial disease; CVD—cardiovascular disease; WBC—white blood cells count; Hb—hemoglobin; PLT—platelet count; CRP—C reactive protein; ACEI—angiotensin—converting enzyme inhibitor; ARB—angiotensin II receptor blocker; ARNI—angiotensin receptor—nephrilysin inhibitor; MRA—mineralocorticoid receptor antagonist; ASA—acetylsalicylic acid.

**Table 5:** Results of multivariate analyses, possible predictors of hyponatremia and hypernatremia.

Characteristics	Coefficient	SE	CI-Min	CI-Max	*p*	OR	CI95 Lower	CI95 Upper	*p*
Predictors of hyponatremia									
Age	0.02	0.00	0.01	0.03	0.0017	1.02	1.01	1.03	0.0017
HR	0.01	0.00	0.00	0.02	0.0177	1.01	1.00	1.02	0.0177
Consciousness	0.17	0.19	−0.19	0.54	0.3538	1.19	0.82	1.72	0.3538
Hypertension	−0.27	0.19	−0.64	0.09	0.1458	0.76	0.53	1.10	0.1458
DM	0.34	0.16	0.02	0.66	0.0376	1.40	1.02	1.93	0.0376
ACEI/ARB/ARNI	0.42	0.18	0.08	0.76	0.0170	1.52	1.08	2.15	0.0170
Beta-blockers	0.01	0.17	−0.33	0.36	0.9400	1.01	0.72	1.43	0.9400
Diuretics	−0.04	0.19	−0.42	0.34	0.8228	0.96	0.66	1.40	0.8228
CRP	0.00	0.00	0.00	0.00	0.0045	1.00	1.00	1.00	0.0045
Creatinine	0.08	0.05	−0.02	0.18	0.0987	1.09	0.98	1.20	0.0987
CVD	0.12	0.17	−0.22	0.46	0.4904	1.13	0.80	1.58	0.4904
Predictors of hypernatremia									
Age	0.02	0.01	0.00	0.03	0.051	1.02	1.00	1.03	0.0510
SBP	−0.02	0.01	−0.04	0.00	0.026	0.98	0.96	1.00	0.0263
DBP	0.02	0.01	0.00	0.05	0.090	1.02	1.00	1.05	0.0896
Consciousness	2.14	0.29	1.58	2.71	0.000	8.54	4.83	15.09	0.0000
Anticoagulants	0.44	0.32	−0.18	1.06	0.163	1.55	0.84	2.89	0.1629
CRP	0.00	0.00	0.00	0.01	0.028	1.00	1.00	1.01	0.0277
CVD	−0.10	0.29	−0.67	0.47	0.728	0.90	0.51	1.60	0.7279

HR—heart rate; DM—diabetes mellitus; ACEI—angiotensin—converting enzyme inhibitor; ARB—angiotensin II receptor blocker; ARNI—angiotensin receptor—nephrilysin inhibitor; CRP—C reactive protein; CVD—cardiovascular disease; SBP—systolic blood pressure; DBP—diastolic blood pressure.

## Data Availability

The datasets used and/or analyzed during the current study are available from the authors upon reasonable request.
